# An algorithm to predict phenotypic severity in mucopolysaccharidosis type I in the first month of life

**DOI:** 10.1186/1750-1172-8-99

**Published:** 2013-07-09

**Authors:** Sandra DK Kingma, Eveline J Langereis, Clasine M de Klerk, Lida Zoetekouw, Tom Wagemans, Lodewijk IJlst, Ronald JA Wanders, Frits A Wijburg, Naomi van Vlies

**Affiliations:** 1 Department of Pediatrics and Amsterdam Lysosome Centre “Sphinx”, Academic Medical Center, University Hospital of Amsterdam, Meibergdreef 9, 1105, AZ Amsterdam, The Netherlands; 2 Laboratory of Genetic Metabolic Diseases, Department of Clinical Chemistry, Academic Medical Center, University Hospital of Amsterdam, Meibergdreef 9, 1105, AZ Amsterdam, The Netherlands

**Keywords:** Mucopolysaccharidosis type I, Iduronidase, Classification, Phenotype, Haematopoietic stem cell transplantation, Residual enzyme activity, Newborn screening, Enzyme replacement therapy, Disease severity

## Abstract

**Introduction:**

Mucopolysaccharidosis type I (MPS I) is a progressive multisystem lysosomal storage disease caused by deficiency of the enzyme α-L-iduronidase (IDUA). Patients present with a continuous spectrum of disease severity, and the most severely affected patients (Hurler phenotype; MPS I-H) develop progressive cognitive impairment. The treatment of choice for MPS I-H patients is haematopoietic stem cell transplantation, while patients with the more attenuated phenotypes benefit from enzyme replacement therapy.

The potential of newborn screening (NBS) for MPS I is currently studied in many countries. NBS for MPS I, however, necessitates early assessment of the phenotype, in order to decide on the appropriate treatment. In this study, we developed an algorithm to predict phenotypic severity in newborn MPS I patients.

**Methods:**

Thirty patients were included in this study. Genotypes were collected from all patients and all patients were phenotypically categorized at an age of > 18 months based on the clinical course of the disease. In 18 patients, IDUA activity in fibroblast cultures was measured using an optimized IDUA assay. Clinical characteristics from the first month of life were collected from 23 patients.

**Results:**

Homozygosity or compound heterozygosity for specific mutations which are associated with MPS I-H, discriminated a subset of patients with MPS I-H from patients with more attenuated phenotypes (specificity 100%, sensitivity 82%). Next, we found that enzymatic analysis of IDUA activity in fibroblasts allowed identification of patients affected by MPS I-H. Therefore, residual IDUA activity in fibroblasts was introduced as second step in the algorithm. Patients with an IDUA activity of < 0.32 nmol x mg^-1^ × hr^-1^ invariably were MPS I-H patients, while an IDUA activity of > 0.66 nmol × mg^-1^ × hr^-1^ was only observed in more attenuated patients. Patients with an intermediate IDUA activity could be further classified by the presence of differentiating clinical characteristics, resulting in a model with 100% sensitivity and specificity for this cohort of patients.

**Conclusion:**

Using genetic, biochemical and clinical characteristics, all potentially available in the newborn period, an algorithm was developed to predict the MPS I phenotype, allowing timely initiation of the optimal treatment strategy after introduction of NBS.

## Background

Mucopolysaccharidosis type I (MPS I, OMIM 252800) is a progressive multisystem lysosomal storage disease (LSD) caused by a deficiency of the lysosomal hydrolase α-L-iduronidase (IDUA, [Genbank NG_008103]), resulting in the accumulation of the glycosaminoglycans heparan sulfate (HS) and dermatan sulfate (DS) in virtually all body tissues [1]. MPS I encompasses a wide phenotypic spectrum, with at the severe end the Hurler phenotype (MPS I-H), which is the most prevalent phenotype, characterized by progressive central nervous system disease in addition to the most prominent somatic manifestations: severe musculoskeletal, pulmonary and cardiac disease, inguinal and umbilical hernias and corneal clouding, all resulting in a significantly reduced life expectancy if left untreated [2-5]. Patients with the intermediate Hurler-Scheie phenotype (MPS I-H/S) are generally reported as having only mild or no cognitive impairment but relatively severe somatic symptoms that limit life expectancy to the 2^nd^ or 3^rd^ decade in the absence of treatment, while the attenuated Scheie phenotype (MPS I-S) is characterized by relatively milder somatic manifestations and a near normal life expectancy [5,6].

Two disease modifying treatment options are currently available in MPS I: hematopoietic stem cell transplantation (HSCT) and intravenous enzyme replacement therapy (ERT) [7]. HSCT can stabilize neurocognitive function, significantly ameliorate the course of several of the somatic symptoms and improve overall survival [8-11]. HSCT is the preferred treatment strategy for patients with a presumed MPS I-H phenotype who are diagnosed before the age of approximately 2.5 years of age [7]. HSCT may also be considered in patients with MPS I-H/S who display progressive neurocognitive involvement [7]. However, although outcomes have improved considerably [12], HSCT still carries a considerable risk for procedure-related morbidity and mortality. Weekly ERT with recombinant IDUA (Laronidase ®) is, therefore, the preferred treatment for patients with the more attenuated phenotypes (MPS I-S and, in general, MPS I-H/S) and ERT was shown to improve respiratory and cardiac symptoms of MPS I and some of the skeletal and joint manifestations, reduce hepatosplenomegaly, and improve the overall quality of life [13-18].

As early initiation of the optimal treatment, either HSCT or ERT, is highly likely to improve clinical outcomes [8,9,11,19], early diagnosis is essential. However, the variable clinical expression and the nonspecific signs and symptoms, in combination with the rarity of the disorder, often lead to a long diagnostic delay [3]. Population newborn screening (NBS), using dried blood spots for detection of MPS I [20-23], is probably the best approach to identify patients at a very young age, thus allowing timely initiation of treatment. The feasibility of inclusion of MPS I in NBS programs is currently studied in several countries [24-26]. Early diagnosis of MPS I through NBS, however, requires early prediction of the phenotype in each MPS I patient to guide decisions on the optimal treatment strategy. To date, assessment of the phenotype is generally based on signs and symptoms at clinical presentation and age of disease onset [15], as genotype is often not informative [27,28]. A recent study, initiated by our group, revealed a lack of consensus between experts on the assessment of phenotypic severity using a scale from 0 to 10, based solely on signs and symptoms at presentation [29]. This may be even more complicated within the scope of a NBS program, as patients may still lack many of the characteristic symptoms on which phenotyping is currently based. In addition to clinical and genetic characteristics, biochemical predictors have been sought to distinguish between phenotypes, but studies so far have been unsuccessful [30,31], except for one study by Fuller *et al*. [32]. The assay reported in this latter study, however, is rather complex and requires the availability of specific antibodies which renders it difficult to implement in other laboratories.

As NBS for MPS I may be implemented in several countries within the near future, there is an urgent need for a tool which allows reliable prediction of the phenotype within the first months of life. Here we present an algorithm for early determination of phenotypic severity in patients with MPS I diagnosed through NBS, combining mutational analysis, determination of residual enzyme activity in cultured skin fibroblasts and clinical characteristics that are apparent within the first month of life. This algorithm may allow separation of those MPS I patients who will benefit from HSCT at an early age from those that will optimally benefit from an early start of ERT.

## Methods

### Outline of the prediction algorithm

We decided to design our algorithm on the separation of two distinct phenotypic categories based on the indications for treatment: patients with MPS I-H, who will benefit from early HSCT and more attenuated non-MPS I-H patients (MPS I-H/S and S), who will benefit from early start of ERT and for whom HSCT is, in general, not considered the optimal treatment strategy [7]. To this aim, we collected data that can all be assessed within the first month of life and might be related to the phenotypic severity: genotype, residual enzymatic activity and GAG storage in cultured skin fibroblasts and clinical signs and symptoms that may become apparent in the first month of life.

### Patients

Thirty patients with MPS I, who were diagnosed and treated in the Academic Medical Centre, Amsterdam, the Netherlands, were included in this study. All patients were classified into three categories (MPS I-H, MPS I-H/S and MPS I-S) by one of the authors (FAW), a clinician experienced in the diagnosis and treatment of lysosomal storage disorders including MPS I. Classification was based on the clinical signs and symptoms at diagnosis and the clinical course of the disease, and not on genotype, biochemical variables or clinical signs in early life.

### Mutation analysis

Mutation analysis had been performed in all 30 patients by standard procedures within the scope of the normal diagnostic workup at our centre. Missense, nonsense, splice site mutations, insertions and deletions were identified. Based on literature [10,27,33-41], potentially discriminating genotypes were identified.

### Residual IDUA activity

#### Fibroblasts

Fibroblast cultures were available from 18 of the 30 patients included in this study. Informed consent for the use of fibroblasts for these studies was obtained from all patients or parents.

#### Cell culture

To remove bovine IDUA activity from the Fetal Bovine Serum (FBS), FBS was inactivated by incubation at 56°C for 30 minutes before use. Patient and control fibroblasts were grown in Dulbecco’s Modified Eagle's Medium supplemented with 10% inactivated FBS and 100 U × ml^-1^ penicillin, 100 μg × ml^-1^ streptomycin and 250 μg × ml^-1^ amphotericin in a humidified atmosphere containing 5% CO_2_ at 37°C. Fresh medium was added every 2 weeks. After culture for 1, 2, 4, 6, 8 or 10 weeks postconfluency, the medium was removed, cell layers were washed with phosphate buffered saline (PBS) and harvested. Cell pellets were washed once with PBS, twice with 0.9% NaCl and stored at −20°C until analysis.

#### IDUA activity analysis

The generally used method to measure residual IDUA activity, using 4-methylumbelliferyl-α-L-iduronide (Glycosynth Ltd., Warrington, Cheshire, England) as substrate [42,43], was optimized by varying the quantity of cell lysate, time of incubation and amount of substrate in order to accurately determine very low enzyme activities in MPS I patient fibroblasts.

Cells were resuspended in PBS and disrupted by sonification using a Vibra Cell sonicator (Sonics & Materials Inc., Newtown, CT, USA). Protein concentration was measured in whole cell lysates as described by Lowry *et al*. [44]. 20 μl of cell lysate was added to 4-methylumbelliferyl-α-L-iduronide in 0.1 M sodium formiate buffer, with a final volume of 60 μl, a final 4-methylumbelliferyl-α-L-iduronide concentration of 1mM and a final protein concentration of 0.5 mg × ml^-1^. After 2 hours of incubation at 37°C, the reaction was stopped by addition of 1440 μl 0.2 M sodium carbonate/glycine buffer, pH 10.5. Released 4-methylumbelliferone was measured fluorometrically with an excitation wavelength of 366 nm and an emission wavelength of 442 nm using a Perkin Elmer LS45 fluorescence spectrometer (Perkin Elmer, Waltham, MA, USA). IDUA activity in each sample was calculated using a calibration curve of 4-methylumbelliferone (Glycosynth Ltd., Warrington, Cheshire, England). All enzyme activity assays were performed in duplicate and repeated at least once in independent cell cultures. Earlier experiments in our laboratory, using control fibroblasts, showed an intra-assay variation of 1.4% and an inter-assay variation of 18.5%. To control for this relatively large inter-assay variation, with each performed IDUA assay, IDUA activity was simultaneously determined in at least 4 other previously analyzed cell lines, to make sure that the results from different experiments could be reliably compared.

### Glycosaminoglycan (GAG) analysis in fibroblasts

Levels of HS and DS were determined as described previously [45] with minor modifications. GAGs in 25 μg of fibroblast lysate (prepared as described for the IDUA activity analysis) were enzymatically digested into disaccharides and as a final deproteination step samples were loaded on an Amicon Ultra 10 kD centrifugal filter (EMD Millipore, Billerica, MA, USA) (instead of a 30kD filter), and centrifuged at 14.000 *g* for 30 minutes on 25°C.

### Clinical characteristics

There are currently only few data published on clinical signs and symptoms in MPS I patients at birth or within the first month of life [46,47]. Therefore, we decided to study the absence or presence of 14 clinical signs and symptoms (Table 1) which are reported as early presenting symptoms in MPS I [46-49]. As several MPS I-H-related clinical characteristics develop only later in life, such as developmental delay and stunted growth, these were not included in our study. If information on certain clinical signs was not available in the charts, parents of the patients were contacted by telephone and additional information was added to the information retrieved from the charts. All characteristics were scored as absent, present, or data not available (Table 2). Clinical characteristics from patients born before 37 weeks of gestational age were excluded, as symptoms related to prematurity, such as respiratory complications or increased occurrence of inguinal hernias may be confounding.

**Table 1:** Clinical characteristics scored in the patient cohort

**Clinical characteristic**	**Number of patients in whom information was available**	**p-value**
**Respiratory manifestations**		
Signs and symptoms of upper respiratory tract obstruction	19	<0.01
**Herniae**		
Umbilical hernia	18	0.6
Inguinal hernia	19	<0.05
**Organomegaly**		
Hepatomegaly	1	X
Splenomegaly	1	X
**Musculoskeletal manifestations**		
Stiff joints/contractures	2	X
Kyphosis	1	X
Scoliosis	1	X
Hip dysplasia	1	X
**Other**		
Hearing impairment	3	X
Hydrocephalus	0	X
Cardiomyopathy	0	X
Macrocephaly	1	X
Corneal clouding	0	X

X = insufficient data available for statistical analysis.

**Table 2:** Patient characteristics

**General information**			**Genetic characteristics**		**Biochemical characteristics**	**Clinical characteristics**	
**Patient ID**	**Phenotype**	**Gestational age (weeks + days)**	**Mutation allele 1**	**Mutation allele 2**	**IDUA activity (nmol x mg**^**-1 **^**× hr**^**-1**^**)**	**Upper respiratory tract involvement**	**Inguinal hernia**
1	H	37 + 0	p.W402X	p.W402X	0.31	-	+
2	H	38 + 1	p.W402X	p.Q70X	X	-	+
3	H	40 + 0	p.W402X	p.Q70X	X	X	X
4	H	39 + 6	p.W402X	p.W402X	0.25	+	-
5	H	37 + 5	p.W402X	c.134del12	0.26	X	-
6	H	33 + 6	p.Q70X	p.L218P	0.47	X	X
7	H	33 + 1	p.Q70X	p.L218P	0.44	X	X
8	H	38 + 0	p.Q70X	p.L218P	0.58	+	X
9	H	37 + 2	p.Q70X	p.L218P	X	+	-
10	H	X	p.W402X	p.L218P	X	+	X
11	H	33 + 0	p.L218P	p.L218P	X	X	X
12	H	40 + 0	p.L218P	p.L218P	X	+	-
13	H	38 + 3	p.W402X	p.L218P	0.43	+	-
14	H	41 + 1	p.A367E	c.1650del117	X	+	+
15	H	38 + 1	c.494-1G > A	c.494-1G > A	0.23	X	+
16	H	X	p.H425fs	p.H425fs	0.32	X	X
17	H	40 + 0	p.W402X	p.W402X	X	X	X
18	H/S	40 + 0	p.W402X	p.R505G	0.77	+	-
19^1^	H/S	38 + 0	p.W402X	p.N348K	0.92	-	-
20^1^	H/S	37 + 1	p.W402X	p.N348K	0.95	-	-
21	H/S	41 + 2	p.L218P	p.D315Y	0.35	-	-
22^2^	H/S	37 + 0	p.P533R	p.P533R	X	-	-
23^2^	H/S	37 + 0	p.P533R	p.P533R	2.43	-	-
24	S	40 + 0	p.W402X	p.R383H	1.05	-	-
25^3^	S	40 + 0	p.Q70X	p.R383H	1.17	X	X
26^3^	S	40 + 0	p.Q70X	p.R383H	X	X	X
27	S	X	p.A327P	p.R383H	1.70	X	X
28^4^	S	40 + 5	c.474-2A > G	p.R383H	X	-	-
29^4^	S	40 + 5	c.474-2A > G	p.R383H	1.61	-	-
30	S	40 + 0	c.474-2A > G	p.R383H	X	-	-

H = Hurler, H/S = Hurler/Scheie, S = Scheie.

+ = present, - = absent, X = data not available or excluded based on prematurity.

^1,3,4^: siblings. ^2^: homozygous twins.

Characteristics that showed a significant difference between MPS I-H and MPS I-non Hurler patients were considered distinguishing and considered for the prediction algorithm.

### Statistics

Statistical analysis was performed using the SPSS Statistics software version 19 (IBM Corp., Armonk, NY, USA). Nonparametric ranking statistics (Mann–Whitney-U tests) were used to analyze the relationship between the assigned MPS I phenotypes (MPS I-H versus non-MPSI-H) and residual IDUA activity and GAG levels in fibroblasts. The most efficient cut-off values to discriminate between MPS I phenotypes based on IDUA activity were identified using receiver operating characteristic (ROC) curve analysis. True positive rates (sensitivity) were plotted against false positive rates (1-specificity) for all classification points, and p-values were calculated for the area under the curve.

Differences between the phenotypic groups (MPS I-H versus non-MPS I-H) in the frequency of specific mutations or clinical characteristics were assessed either by Fisher’s exact test (dichotomous variables) or Mann–Whitney-*U* test (numeric variable).

A three-step algorithm was designed and discrimination of the three phases in the flow chart was assessed separately by calculating sensitivity and specificity. This was also done for the algorithm as a whole, as a way to perform internal validation.

All p-values were based on two-sided testing and differences with p-values < 0.05 were considered statistically significant.

## Results

For all 30 patients with MPS I included in this study, information on at least one potentially predictive criterion (genetic, biochemical or clinical) was available.

### Mutation analysis

Based on the literature [10,27,33-41], a list of 25 mutations, which have been shown to reliably predict a Hurler phenotype when patients are homozygous or compound heterozygous for these mutations, was constructed (Table 3). In our cohort, the association of these mutations with MPS I-H could be confirmed for the mutations p.Q70X, p.W402X, p.L218P and c.134del12.

**Table 3:** **Mutations described as MPS I**-**Hurler associated in literature**

**Nonsense**	**Reference**	**Other**	**Reference**
p.W41X	[40]	p.G51D	[33,38]
p.Y64X	[37]	c.134del12	[10,34]
p.Q60X	[27]	p.G208V	[27]
p.Q63X	[37,39]	p.L218P	[27,34]
p.Q70X	[33,37]	p.A327P	[27,33]
p.P201X	[33]	p.T366P	[37]
p.E274X	[37]	c.704ins5	[37]
p.Q310X	[37]	c.1190-1G > A	[41]
p.Y343X	[37,39]		
p.W402X	[33,37]		
p.E404X	[37,40]		
p.Q561X	[36]		
p.Y581X	[35]		
p.Q584X	[37]		
p.R619X	[27]		
p.R621X	[34]		
p.W626X	[40]		

As mutations that have been associated with the more attenuated phenotypes [30,37,50] may be more susceptible to the effects of modifying polymorphisms in other genes [50], these mutations were not included in our algorithm. Using the list of predictive mutations in Table 3 in our group of 30 patients, a specificity of 100% for prediction of the MPS I-H phenotype and a sensitivity of 82% was calculated. The list of predictive mutations was integrated as first step in the prediction algorithm.

### Biochemical analyses

#### Residual IDUA activity

**Figure 1:**
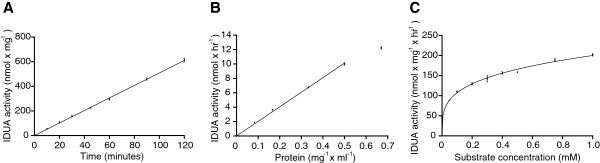
**IDUA activity analysis. (A)** Time dependence, using 1 mM substrate and 0.17 mg × ml^-1^ protein. **(B)** Protein dependence, using 1 mM substrate and an incubation time of 60 minutes. **(C)** Substrate titration, using 0.17 mg × ml^-1^ protein and an incubation time of 60 min.

Figure 1 shows that IDUA enzyme activity was linear up to at least 120 minutes of incubation time (Figure 1A) and 0.5 mg × ml^-1^ final protein concentration (Figure 1B). Based on these findings, we selected these conditions for subsequent studies. Substrate titrations were performed (Figure 1C) and although maximal enzyme activity was not reached, subsequent activity measurements were performed using a final substrate concentration of 1 mM. This concentration resulted in a 45% increase in activity, as compared to the commonly used substrate concentration [42,43].

**Figure 2:**
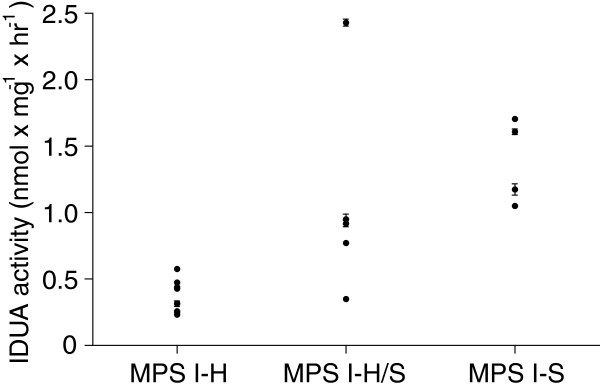
**Residual IDUA activity in MPS I fibroblasts of patients with different phenotypes.** IDUA activity for each patient is reported in Table 1. IDUA activity was measured in duplicate in each sample. All experiments were at least repeated once in independent cell cultures, the results of one representative experiment are shown. All values are mean ± standard deviation. Control range: 101–270 nmol × mg^-1^ × hr^-1^.

IDUA activity was determined in human skin fibroblast cell lines from the 18 MPS I patients (Table 2 and Figure 2) of whom cell lines were available. Analyses were performed a week after the cells had reached confluency.

**Figure 3:**
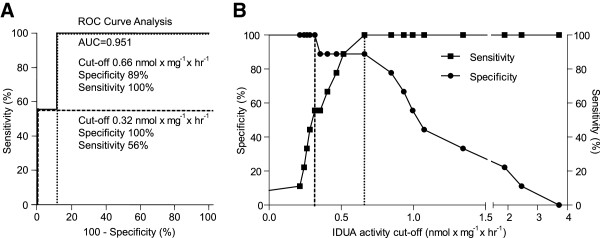
**ROC curve analysis. (A)** ROC curve of IDUA activity for discrimination between MPS I-H and non MPS I-H. **(B)** Sensitivity and specificity for IDUA activity cut-off levels to discriminate between MPS I-H and non MPS I-H. Dashed lines represent chosen cut-off levels of 0.32 and 0.66 nmol × mg^-1^ × hr^-1^ IDUA activity.

Residual IDUA activity in MPS I cell lines ranged from 0.23-2.43 nmol × mg^-1^ × hr^-1^ (Table 2), which is less than 2.5% of the activity found in control fibroblasts (control range: 101–270 nmol × mg^-1^ × hr^-1^). MPS I-H fibroblasts could be completely discriminated from the MPS I-S fibroblasts (p < 0.01) based on IDUA activity. Most MPS I-H/S fibroblasts had an intermediate IDUA activity (Figure 2). The diagnostic accuracy of the IDUA assay in differentiating MPS I-H from non-H MPS I patients showed an area under the ROC curve of 0.951 (p < 0.001, Figure 3A), indicating a good discrimination. Two cut-off values were calculated, resulting in three categories of enzyme activity: an IDUA activity of < 0.32 nmol × mg^-1^ × hr^-1^ identified MPS I-H fibroblasts with a specificity of 100% (sensitivity 56%), as shown in Figure 3B. This was regarded as the lower threshold, as only MPS I-H patients were found below this level of activity. Furthermore, 100% sensitivity (specificity 89%) was reached at a cut-off value of 0.66 nmol x mg^-1^ × hr^-1^ enzymatic activity to discriminate MPS I-H fibroblasts from cell lines of non-H MPS I patients (Figure 3B). Subsequently, this was set as the upper threshold; no MPS I-H fibroblasts had an enzyme activity higher than 0.66 nmol × mg^-1^ × hr^-1^.

The same sensitivity for discrimination of phenotypes was obtained when cells were cultured for 2, 4, 8 or 10 weeks postconfluency. With increasing culture time, however, residual enzyme activity in all fibroblast cell lines decreased, as compared to cells cultured for 1 week postconfluency (results not shown).

#### Heparan sulfate and dermatan sulfate levels in fibroblasts

No significant differences were seen between MPS I-H fibroblasts and non-MPS I-H cells in total HS and DS or in the levels of individual disaccharides (results not shown).

### Clinical characteristics

Information on clinical signs and symptoms in the first month of life was available for 23 patients (Table 2). 3 patients, however, were excluded from the analysis because they were born at a gestational age < 37 weeks. A significant difference between the incidence of signs and symptoms of upper respiratory tract obstruction (p = 0.005) and inguinal hernia (p = 0.033) was found between MPS I-H patients and non-MPS I-H patients.

### Prediction algorithm

Mutation analysis was integrated as the first step in the prediction algorithm (specificity 100%, sensitivity 82%) and IDUA activity was chosen as the second step. A cut off value of < 0.32 nmol × mg^-1^ × hr^-1^ was used to identify MPS I-H patients. An IDUA activity of >0.66 nmol × mg^-1^ × hr^-1^ identified non-MPS I-H patients. Patients with IDUA activity between 0.32-0.66 nmol × mg^-1^ × hr^-1^ were further classified by the presence of either one of the associated clinical characteristics (sensitivity 100%, specificity 100%).

**Figure 4:**
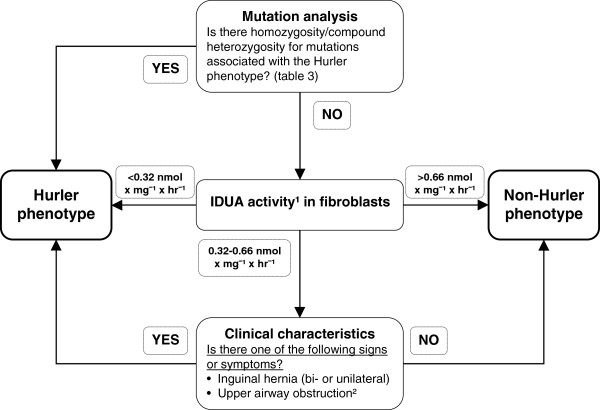
**Algorithm for assessment of phenotypic severity in MPS I patients.** Mutational analysis, residual IDUA activity and clinical characteristics present before the age of 1 month are combined. ^1^Measured as described in this article. ^2^Upper airway obstruction included the following symptoms: excessive snoring during sleep, continuously runny nose, obstructive sleep apneas, feeding difficulties due to obstructed nasal breathing.

This resulted in a sensitivity and specificity of 100% for the complete prediction algorithm. The flow chart for the prediction algorithm is shown in Figure 4.

## Discussion

Here we present an algorithm, based on the combination of mutation analysis, residual IDUA activity and clinical signs and symptoms during the first month of life, which may allow early, sensitive and specific prediction of the phenotype in MPS I patients diagnosed through NBS. Such an algorithm can be essential as the decision to implement NBS for MPS I will depend, at least in a number of countries, on the feasibility to decide on the optimal treatment strategy at an early age. NBS for MPS I is of high interest as early initiation of treatment, i.e. either HSCT for patients who will develop a MPS I-H phenotype and ERT for the non-MPS I-H patients, likely improves the disease outcome [8,9,12,19,51], and early diagnosis on clinical recognition can be very difficult.

To date, more than 200 different mutations in the IDUA gene have been reported [52], and this genetic heterogeneity partially explains the phenotypic variability in MPS I. For most of the mutations no clear genotype-phenotype correlation is known. However, some mutations have been found to reliably predict a severe disease phenotype [10,27,33-41]. This was confirmed in our cohort for the mutations p.Q70X, p.W402X, p.L218P and c.134del12. Therefore, mutation analysis was included as the first step in the algorithm to predict MPS I phenotype. Several missense mutations, such as the p.R383H and p.R89Q mutations, are generally reported in association with more attenuated disease [30,37,50]. We did not include these latter mutations in our algorithm, however, because the effect of attenuated mutations might vary due to novel combinations of mutations, polymorphisms in other genes or environmental factors [50]. Other mutations present in our cohort were also not incorporated in the algorithm because of functional heterogeneity (e.g. the same mutations seem to have a different effect on phenotypic severity) in earlier studies, such as the mutations p.P533R and c.474-2A > G [27,33,37]. Studies on genotype-phenotype correlations in large cohorts, focusing on allelic combinations of rarer mutations, could further improve the predictive power of this first step in our algorithm. Currently, rapid mutation analysis of the *IDUA* gene may not be available to all centers diagnosing MPS I. However, the fast technological advancements for gene sequencing will result in more universal access to mutation analysis, allowing reliable results within 4 weeks after diagnosis for most patients and applicability of the algorithm presented in this study.

It is highly likely that the introduction of NBS for MPS I will result in the identification of many novel mutations with unknown phenotypes. Therefore, a tool for prediction of phenotypic severity within the scope of NBS needs to include other variables. We found that the concentrations of HS and DS and of the individual disaccharides in cultured fibroblasts did not correlate with the phenotype. In contrast, Fuller *et al*. demonstrated that the levels of specific HS and DS derived trisaccharides in patient fibroblasts could discriminate between MPS I patients with and without neurological involvement [32]. In that study, only levels of short chain HS and DS oligosaccharides were measured, while the HS and DS derived disaccharides detected in our study originate predominantly from relatively larger HS and DS chains [45]. Possibly, fibroblasts from patients with neurological involvement store more short GAGs chains, as compared to patients without neurological symptoms, but similar levels of larger HS and DS oligosaccharides, which hinders discrimination between these phenotypes using our GAG analysis.

Analyses of IDUA activity in fibroblasts or leukocytes is generally used as the confirmatory step in MPS I diagnosis. However, the most commonly used method, though sensitive for diagnostics [42,43], is not sensitive enough to reliably discriminate between the different MPS I phenotypes. A study in a cohort of 13 MPS I patients [32], where the IDUA protein was first captured using antibodies followed by enzymatic studies, showed clear discrimination between patients with and without neurological involvement. This method, however, makes use of specific anti-IDUA antibodies which are not commercially available, making this assay difficult to implement in other laboratories. In addition, specific putative mutations might result in a loss of epitopes, obstructing capture of the protein and thus interfering with the analysis. For this reason, we optimized the 4-methylumbelliferyl-α-L-iduronide IDUA activity assay to provide a method that may be more generally applicable. A higher concentration of substrate, independently reported by others to improve the accuracy of the IDUA assay in a recent study [53], combined with a longer incubation time and increased amount of protein, resulted in accurate measurement of very low enzyme activities, as seen in MPS I patients [43]. As these are minor changes to the commonly used IDUA activity analysis protocol, but very important to accurately determine very low IDUA activities, we feel that most laboratories will be able to implement this protocol after the necessary validation steps. Interestingly, a very narrow range of low IDUA activities is responsible for a broad range of clinical presentations in MPS I patients, as IDUA activity in all MPS I fibroblasts was less than 2.5% of the activity measured in healthy control fibroblasts. Despite this small range of IDUA activities, cut-off values could be calculated using ROC curve analysis to differentiate between MPS I Hurler and non-Hurler fibroblasts.

Measurement of residual IDUA activity could not fully differentiate between phenotypes of patients with an activity in the range of 0.32-0.66 nmol × mg^-1^ × hr^-1^, as one MPS I-H/S cell line had an IDUA activity in this range. Although HSCT may be considered in some MPS I-H/S patients with neurocognitive involvement [7], this is not common practice. Therefore, the algorithm was improved by inclusion of potentially discriminating clinical characteristics early in life.

Of the 14 clinical characteristics studied, the presence of two were found to differ significantly between MPS I-H and non-MPS I-H MPS I patients: presence of inguinal hernia and the presence of signs and symptoms of upper airway obstruction. Including clinical characteristics in the algorithm resulted in complete differentiation between MPS I-H patients and patients with more attenuated phenotypes. Another clinical characteristic that may differentiate between MPS I-H patients and more attenuated patients is probably the severity of dysostosis multiplex, a collection of radiographic abnormalities resulting from defective endochondral and membranous growth throughout the body seen in mucopolysaccharidoses. Especially thoraco-lumbar kyphosis before the age of one month, might be a very sensitive and specific symptom for MPS I-H [54,55]. However, early kyphosis is often initially not recognized by parents and caregivers and could therefore not be included in this model, which is based on retrospective analysis of clinical data.

Our study has some limitations. Firstly, due to the ultra-orphan nature of the disease, the proposed algorithm is validated in only a relatively small number of patients (n = 30). Validation in other cohorts of patients needs to be performed to further determine its value. Secondly, our study includes a retrospective analysis of signs and symptoms during the first month of life. This may result in a recall bias, as both parents and investigators knew the phenotype of the patients. To address this, only characteristics that could be clearly distinguished and are often well documented in the newborn period were used for this algorithm. Thirdly, the prevalence of mutations firmly associated with certain phenotypes differs between regions around the world [37]. Therefore, positive and negative predicting values of the proposed algorithm may differ between countries and this needs to be further investigated. Also, as new mutations will be detected once NBS for MPS I has been introduced, a prediction algorithm including mutation analysis needs to be continuously adjusted and improved. Likewise, NBS will allow for further investigation on the predictive value of certain clinical signs such as early kyphosis, which could not be included in this study.

As a result of future studies, the algorithm might be adapted to also to differentiate between MPS I-H/S patients with and without neurocognitive involvement. The improved outcome of HSCT, in combination with increasing knowledge on the risk for neurocognitive decline in a subset of MPS I-H/S patients, may result in a shift in treatment protocols, with HSCT as treatment of choice for this group of patients [7].

With the phenotypic prediction algorithm presented here, we hope to provide the basis for a tool to reliably predict phenotype in the majority of MPS I patients diagnosed through NBS. Prospective studies could result in inclusion of additional predictive factors and improvement of the prediction algorithm.

## Conclusion

Using genetic, biochemical and clinical characteristics, which can all be studied within the first month of life, an algorithm was developed for accurate prediction of the phenotype at an early age in MPS I patients. Such an algorithm allows timely initiation of the optimal treatment strategy, thus improving disease outcome. With the future launch of NBS programs for MPS I, patients will not have developed all characteristic signs and symptoms currently used for assessment of the phenotype, making a prediction algorithm for early assessment of phenotypic severity indispensable.

## Abbreviations

DS: Dermatan sulfate; ERT: Enzyme replacement therapy; FBS: Fetal bovine serum; GAG: Glycosaminoglycan; HS: Heparan sulfate; HSCT: Haematopoietic stem cell transplantation; IDUA: α-L-iduronidase; IRDS: Infant respiratory distress syndrome; LSD: Lysosomal storage disease; MPS I: Mucopolysaccharidosis type I; MPS I-H: MPS I-Hurler; MPS I-H/S: MPS I-Hurler/Scheie; MPS I-S: MPS I-Scheie; NaCl: Sodium chloride; NBS: Newborn screening; PBS: Phosphate buffered saline; ROC: Receiver operating characteristic.

## Competing interests

The authors declare that they have no competing interests.

## Authors’ contributions

SDKK, EJL, NvV: Conception and design, data acquisition, analysis, and interpretation, manuscript draft and revision. CdK: data acquisition and analysis, manuscript draft. LZ: conception and design, manuscript revision. TW: data analysis, manuscript revision. LIJ, RJAW: conception and design, manuscript revision. FAW: Conception and design, manuscript draft, manuscript revision. All authors read and approved the final manuscript.

## Acknowledgements

We thank W. Kulik and H. van Lenthe for helpful discussions and technical assistance.
